# Larsen Syndrome and Associated Spinal Deformities

**DOI:** 10.7759/cureus.41655

**Published:** 2023-07-10

**Authors:** Angeliki Siafaka, Stavros Angelis, Maria Piagkou, Alexandros Apostolopoulos, Theodore Troupis, Dimitrios Filippou

**Affiliations:** 1 Anatomy Department, National and Kapodistrian University of Athens, Athens, GRC

**Keywords:** spine deformities, kyphoscoliosis, congenital joint dislocations, genetic disorder, larsen syndrome

## Abstract

Larsen syndrome is a rare genetic disorder that affects the connective tissue within the body. The present narrative review aims to examine the genetic basis of Larsen syndrome, clarify its symptoms, and define all the existing therapeutic approaches. A comprehensive search was performed in the PubMed database. Inclusion criteria considered molecular and clinical studies, management and surgical treatment of related deformities, case reports of patients with the syndrome, reviews of the associated anomalies, articles whose full text is available in PubMed, and articles published in the English language. Larsen syndrome is caused by mutations in the *FLNB* gene, which encodes the cytoskeletal protein filamin B, crucial in the development of the skeleton. Symptoms include joint dislocations, characteristic facial features and anomalies of the spine. Larsen syndrome may be conservatively treated initially, although surgical intervention is usually required. Various surgical techniques, including posterior spinal fusion, anterior decompression, circumferential arthrodesis, and single-stage 360° fixation, have been proposed along with growth-sparing procedures. Preoperative and postoperative care and education ensure optimal results. Further research is needed to identify novel therapeutic modalities for this condition.

## Introduction and background

Larsen syndrome (LRS; Online Mendelian Inheritance in Man {OMIM} #150250) is an uncommon inherited disorder featuring numerous joint dislocations, distinct facial characteristics, and spinal anomalies. Its root cause lies in a mutation of the *FLNB* gene (OMIM:603381), responsible for producing the protein filamin B. The syndrome is typically inherited via either autosomal dominant or recessive patterns and can be diagnosed based on clinical or radiographic indications, along with genetic testing. Additional, potentially fatal complications may arise, such as cardiac malfunctions, brain irregularities, or respiratory issues [1]. The onset of symptoms usually begins in early childhood or adolescence [2]. This condition is classified as osteochondrodysplasia [3,4].

Larsen syndrome was initially described in 1950 by the Danish pediatrician Dr. Oluf Daniel Larsen [5-8], and its prevalence remains understudied, with an estimated occurrence rate of 1 in every 100,000 to 200,000 live births [2,6]. Gender does not affect the incidence, and the syndrome affects all individuals regardless of racial and ethnic background [3].

## Review

Methods

This narrative review aims to examine the genetic basis of Larsen syndrome, along with its associated symptoms and the available management options for the complex deformities that it presents. A comprehensive search of the PubMed database was conducted utilizing the search term "Larsen syndrome AND spine deformities" in March 2023. The search was limited to studies published between 1977 and 2022, resulting in 36 articles. An additional search was conducted using the keywords "Larsen syndrome," "spinal diseases," "spine deformities," and "spine anomalies," which generated 30 articles. After eliminating duplicates (n=22), 44 articles remained for assessment in this review.

The inclusion criteria encompassed (a) molecular and clinical studies, (b) scholarly articles pertaining to the overall diagnosis and management of the condition, (c) case reports of patients with rare Larsen syndrome symptoms, (d) reviews of anomalies associated with Larsen syndrome, (e) articles, the full text of which is available in PubMed, and (f) articles published in the English language.

**Figure 1:**
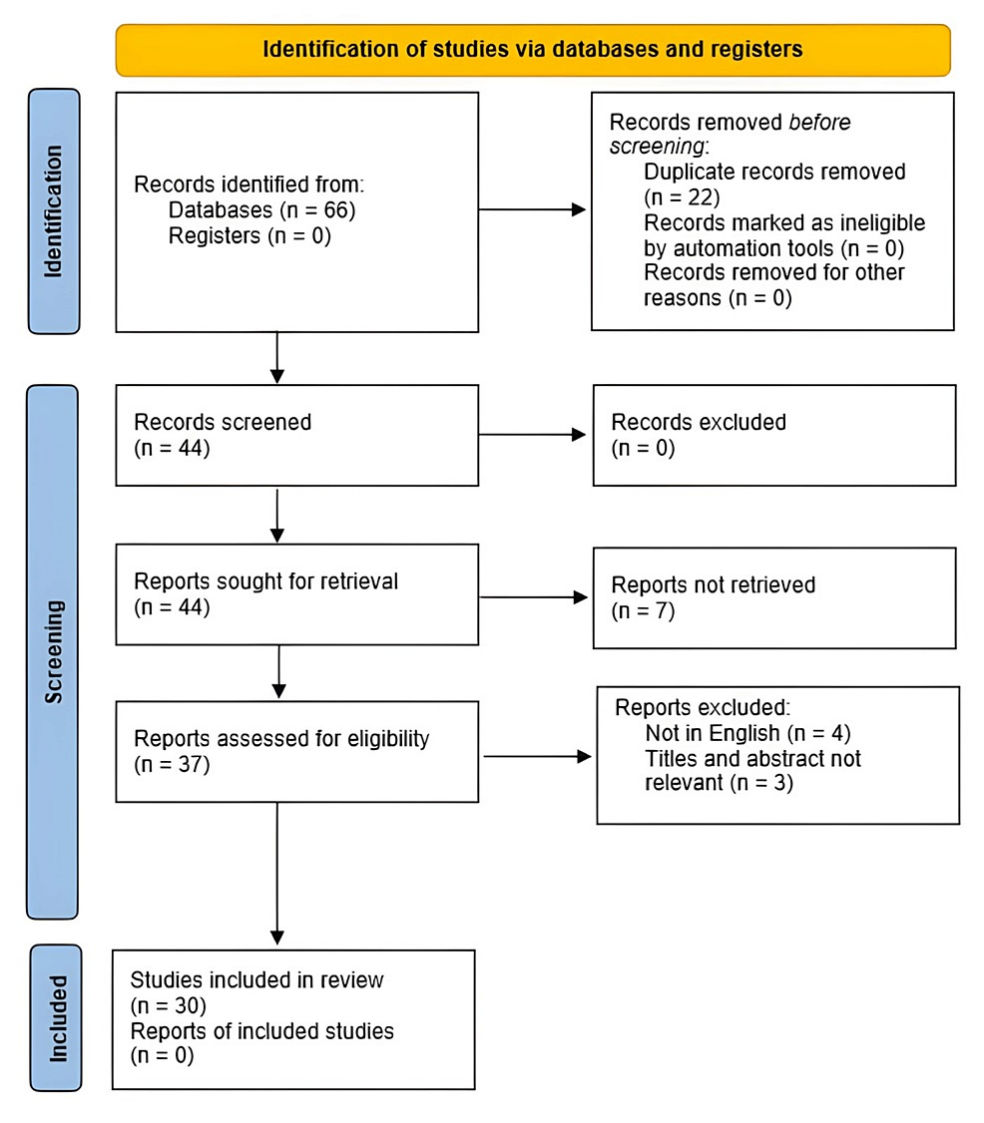
Flow diagram presenting the method based on which the articles for the paper were accumulated

After applying the above criteria, 14 articles were excluded, resulting in 30 articles being utilized for this review (Figure 1).

Results and discussion

Genetic Basis

Larsen syndrome (LRS; OMIM #150250) is a genetic disorder that impacts connective tissue within the body. The underlying cause of this condition is attributed to clusters of missense mutations or small inframe deletions found in the *FLNB* gene (OMIM:603381), which is situated on chromosome 3p14.3 [3,4,9-13]. The *FLNB* gene is composed of 46 exons [14], two calponin homology domains at the N-terminal (CH1, CH2), which create an actin-binding domain (ABD), and 24 structurally homologous repeats that are linked by two hinge regions (hinge-1, hinge-2) situated between repeats 15 and 16 as well as repeats 23 and 24 [3,12-14]. It is noteworthy that the mentioned mutations' number and distribution are non-random. Rather than being dispersed throughout the gene, the mutations are concentrated within specific regions, namely the actin-binding domain, the CH2 domain, and filamin repeats 2, 13, 14, 15, 17, and 23 [3,12]. This pattern of localization suggests the criticality of these regions in shaping the protein's structure and, consequently, its function. Encoded by the *FLNB* gene, the filamin B cytoskeletal protein comprises 2602 amino acids [12] and holds a vital role in skeletal development. Through dimer formation and actin binding, filamin B links actin filaments and forms a pliable structure that interfaces with adhesive receptors on cellular membranes [12,13]. Henceforth, filamin B facilitates the communication between the cellular membrane and cytoskeleton-a complex of protein fibers responsible for cell shape and locomotion [10]. It supports cytoskeletal proteins such as actin to adapt to structural and growth changes [9]. In addition, recent studies have indicated filamin B's involvement in regulating the shift of connective tissue cells, including chondrocytes, osteoblasts, and fibroblasts, crucial for skeletal growth [12]. Notably, mutations in the FLNB gene can incapacitate filamin B from performing its functions effectively. Specifically, missense mutations in the gene's actin-binding domain have been shown to enhance affinity between filamin B and actin molecules, leading to increased levels of the actin-filamin B complex in the cell [12]. As such, the aforementioned mutation in the FLNB gene induces a gain of function in the resultant protein, leading to one of the variants of Larsen Syndrome, specifically the autosomal dominant variation [12].

In order to investigate the role of filamin B (FLNB) in the human body, a study was conducted whereby mice were genetically modified with a disabled version of the *FLNB* gene. The results showed that less than 3% of the homozygous embryos reached term, which indicates that FLNB plays a pivotal role in proper embryonic development [15]. Interestingly, heterozygous mutant mice, where one copy of the *FLNB* gene was functional and one disabled, did not demonstrate any noticeable distinctions in comparison to their wild-type counterparts. It was found that the FLNB gene was expressed in a variety of cells, with a particular emphasis on endothelial cells and chondrocytes. Furthermore, when FLNB-deficient fibroblasts were compared to wild-type controls, the former exhibited less organized actin filaments and impaired migration ability [15]. The embryos lacking the *FLNB* gene exhibited compromised microvasculature and impaired development of the skeletal structure resulting in severe malformations such as spinal curvature, vertebral body fusion, lack of intervertebral discs, and reduced hyaline matrix in the extremities, thorax, and vertebrae leading to their small size. Their deformities were so severe that they could either not survive beyond four weeks or had to be put down. These results establish the similarity between the impact of a defective FLNB gene in mice and humans experiencing mutations in the FLNB gene, causing skeletal disorders [15].

Several mechanisms have been described regarding how mutations in the FLNB gene (OMIM:603381) may cause skeletal malformations [12]. The initial mechanism involves delayed ossification in the long bone growth plate. Studies on mice with FLNB mutations have revealed a decrease in the proliferation zone and an expansion of the hypertrophic zone, leading to diminished chondrocyte proliferation and delayed ossification. Additionally, *FLNB* variants have been linked to decreased bone mineral density in various bones, including the vertebrae and the neck of the femoral bone [12]. The third mechanism suggests that mutations in this gene may also disrupt muscle fiber differentiation, resulting in weakness and muscular imbalances that can lead to skeletal anomalies like kyphosis and scoliosis. The abnormality in *FLNB* has the potential to impact intervertebral disc ossification through a multifaceted signaling pathway. In mice, the mutation of *FLNB* results in the development of ectopic mineralization in cartilaginous components, which can lead to skeletal deformities such as scoliosis, kyphosis, and fusion. Research has indicated that a loss of filamin B can lead to disturbances in chondrocyte differentiation, reduced proliferation, heightened apoptosis, and impaired growth plate structure [12]. Ultimately, mutations in the *FLNB* gene may inhibit the development of vital blood vessels that provide necessary nutrients to bone formation sites during ossification [12].

Two distinct forms of Larsen syndrome exist, namely autosomal dominant and autosomal recessive [5,6,16]. The former is the most prevalent variation, where an individual inherits one mutated instance of the *FLNB* gene from a parent [3,13]. Among its defining features are dislocations of the joints, craniofacial abnormalities, and malformations of the extremities [3]. On the other hand, the recessive variety manifests when a person receives two anomalous copies of the FLNB gene, one from each parent [3]. However, some cases of recessive Larsen syndrome can also be linked to mutations in genes other than *FLNB*, for example, CHST3 (OMIM:603799) [17]. Patients with this form often exhibit joint dislocations, changes in the vertebrae, normal age range of carpal bones, absence of facial flattening, and pulmonary anomalies [6,17]. While differentiating between the two strains may prove to be a challenging task, the recessive type appears to be more severe [6,18]. It is important to note that the proportion of cases of Larsen syndrome caused by CHST3 deficiency is currently unknown, but it is believed to be higher than previously thought [17]. Additionally, Larsen syndrome may be linked to somatic or germline mosaicism or de novo events [13]. The option of molecular diagnosis/genetic testing may be beneficial in the care and counselling of those affected and their families [13,19]. As a whole, Larsen syndrome remains a genetically and molecularly diverse disorder, and further research is necessary to comprehend its root causes and explore potential treatments [6,13,18-20].

Symptoms and Clinical Presentation

All reported symptoms of Larsen syndrome are included in Table 1.

**Table 1:** Symptoms of Larsen Syndrome

Multiple joint dislocations, particularly in the elbows, wrists, hips, and knees [1,4-8,11-13,16,18,21,22]
Ligamentous laxity [8,16]
Characteristic facial features such as a "dish face" with a saddle nose, hypertelorism (widely spaced eyes), a round face, and a flattened midface [4-9,11-13,16,18,21]
Frontal bossing [5,6,11,16]
Fusion of frontal and parietal bones [18]
Dysgraphia [6]
Low set ears [18]
Short, broad neck with redundant neck skin and subcutaneous tissue [18]
Cleft palate [1,3,7,10,13,16,18,19,21]
Distinctive deformities of the hands and feet, which include clubfoot (talipes equinovarus deformity of the feet) [1,5,6,13,18], supernumerary carpal and tarsal bones and short, broad, spatulate distal phalanges, particularly of the thumb [3,6,7,11-13,16,21]
Rhizomelic shortening of limbs [18]
Congenital metatarsus (primus) varus [8]
Congenital dysplasia of the humerus [21]
Bilateral single palmar creases [18]
Ulnar deviated wrists [8]
Barrel-shaped chest with flared ribs [6]
Pectus excavatum with intercoastal retractions [6]
Airway obstruction due to mobile arytenoid cartilage [1]
Laryngomalacia [5,6,13]
Tracheomalacia [1,3,5,6,10,13]
Bronchomalacia [1,5]
Pulmonary hypoplasia [6,12,18]
Restrictive lung disease [5]
Subglottic stenosis [5]
Cardiac abnormalities such as atrial/ventricular septal defects and mitral, aortic, or tricuspid valve lesions (similar to Marfan syndrome) [1,5,6]
Aortic root dilatation and aortic regurgitation [5,6]
Mitral valve prolapse [6]
Mitral regurgitation [6]
Subaortic stenosis [5,6]
Cardiomyopathy [5,6]
Patent ductus arteriosus [6]
Short stature [3,6,7,17,19]
Late onset of walking [1,16]
Kyphoscoliosis [5,6,8,9,16,17,22]
Cervical kyphosis [1,3-6,8-10,13,16-18,22]
Thoracolumbar kyphosis [1,4,16]
Scoliosis [3,7,8,12,13]
Spondylolisthesis [13]/spondyloptosis [2]
Segmentation anomalies of the vertebrae [4,6,9,16]
Hypoplasia of the vertebral bodies [4-6,13,16,18]
Hemivertebrae [5,13]
Craniovertebral junction instability [4,8]
Atlantoaxial instability [4]
Cervical cord compression/compromise of cervical cord function [5,16]
Myelopathy [4,5,13]
Hypotonia [6,16]
Hearing loss [3,6,12,13]
Osteoarthritis (in older patients) [1]

The primary indicators affiliated with Larsen syndrome encompass joint dislocations, prevalently occurring in the elbows, wrists, hips, and knees [1,4-8,11-13,16,18,21,22], together with distinct facial features such as a "dish face" with a saddle nose, hypertelorism [4-9,11-13,16,18,21], and frontal bossing [5,6,11,16], along with spinal anomalies. Furthermore, Larsen syndrome is customarily associated with unique deformities of the hands and feet, including clubfoot [1,5,6,13,18], supernumerary carpal and tarsal bones, short and broad distal phalanges, particularly of the thumb [3,6,7,11-13,16,21], and limb and long bone dysplasia. According to some, the triptych comprising hypoplasia of the odontoid process, small and bullet-shaped vertebrae bodies, and the typical "dish face" are definitive of Larsen syndrome and lead to its identification [4,8]. In contrast, others assert that the distinctive facial features and anterior knee dislocation are adequate for diagnosing Larsen syndrome [5].

The aforementioned symptoms are frequently accompanied by respiratory and cardiovascular abnormalities. Larsen syndrome-related respiratory anomalies include laryngomalacia [5,6,13], tracheomalacia [1,3,5,6,10,13], and bronchomalacia [1,5], all of which involve excessive airway compliance leading to partial cartilage collapse during respiration. Additionally, individuals with Larsen syndrome may exhibit pulmonary hypoplasia [6,12,18], restrictive lung disease [5], and subglottic stenosis [5]. In Larsen syndrome, cardiovascular anomalies may manifest as atrial/ventricular septal defects, mitral, aortic, or tricuspid valve lesions, and share similarities with Marfan syndrome [1,5,6]. Manifestations can also include aortic root enlargement and aortic regurgitation [5,6], mitral valve prolapse [6], mitral regurgitation [6], subaortic stenosis [5,6], patent ductus arteriosus [6] and cardiomyopathy [5,6]. It is important to consider both respiratory and cardiovascular conditions in the treatment process, as they can pose additional challenges. Assistance from a pulmonologist or cardiothoracic surgeon may be necessary.

Patients affected by Larsen syndrome frequently exhibit spine abnormalities that stem from various underlying deformities, such as hypoplasia of the vertebral bodies, hemivertebrae, or segmentation anomalies of the vertebrae [5]. A particular concern is the presence of deformities in the cervical spine, as it poses a significant risk for cord compression and can result in life-threatening complications [4,8,13]. One of the most severe indications of Larsen syndrome is the development of cervical kyphosis, a condition characterized by abnormal neck curvature, potentially resulting in the impingement of the spinal cord [7,16]. This condition is associated with progressive instability, cervical cord myelopathy, distal muscle weakness/atrophy, and quadriplegia, making it a critical aspect in assessing and managing patients with Larsen syndrome [4,9,13]. It is essential to highlight that in certain cases, cord compression may not transpire at the level of the kyphotic deformity but rather at its adjacent levels, both cranial and caudal [22]. This gradient may result from the possible fusion of the kyphotic vertebrae, further complicating the case [22]. Research indicates that the occurrence of cervical kyphosis in those with Larsen syndrome is roughly 12% [4,9], and the likelihood of myelopathy is almost 15% [13]. Therefore, obtaining cervical spine radiographs immediately after diagnosing Larsen syndrome is pivotal, as early detection of cervical kyphosis and subluxation is imperative to prevent fatalities in these individuals [3]. The utmost critical variant of cervical dislocation in Larsen syndrome is spondyloptosis [2]. Despite the substantial displacement of vertebrae, neurological deficits may be minimal in spondyloptosis due to the destruction or enlargement of the posterior vertebral elements, leading to spinal canal decompression [2]. Nonetheless, there is a dearth of information regarding surgical methods used to treat this condition.

Larsen syndrome can prompt the emergence of scoliosis at an early stage, which can be exceptionally inflexible and necessitates timely intervention [1,3]. Scoliosis in those with Larsen syndrome may lead to an irregularity of the thoracic area, hampering lung expansion and conceivably impacting heart and lung functions [12]. One of the gravest defects mentioned is thoracic insufficiency syndrome, a state where scoliosis-induced spinal rotation distorts the ribcage, decreasing the transverse chest volume. This may provoke a Type IIIb volume depletion deformity of the thorax [1].

People with Larsen syndrome frequently display low muscle tone, or hypotonia, resulting in delays in motor skill acquisition such as walking [1,6,16]. Critical features of this condition are joint laxity and dislocations, particularly an anterior knee dislocation [5,16]. Both hypotonia and these orthopedic concerns contribute to the delayed development of walking and other milestones [16]. Malformations in the middle ear's auditory ossicles frequently result in hearing loss, which is a typical complication [3,6,12,13].

Treatment 

To effectively manage rare congenital diseases like Larsen syndrome, an accurate diagnosis is paramount [1,23]. Once the diagnosis is established, patients require a comprehensive preoperative evaluation to identify potential risks and complications associated with surgery, particularly in relation to musculoskeletal, respiratory, cardiovascular, and neurological systems [1,6]. Given the high prevalence of spine abnormalities in this syndrome, a thorough evaluation of the spine, including screening anteroposterior radiographs, lateral views of extension and flexion, and MRI scans, is crucial [1,6]. Timely screening enables prompt identification and management of major abnormalities, such as cervical kyphosis and myelopathy, preventing severe neurological decline that can pose life-threatening risks for these patients [1,8,10,23,24]. It is imperative to consult with a pulmonologist to evaluate pulmonary status, and a preoperative bronchoscopy to assess for airway malacias, such as laryngomalacia, tracheomalacia, and bronchomalacia may be necessary [1]. In instances of a hypermobile airway or malacia, anesthesiology consultation is recommended, as anesthesia administration may present complications [1,5,6]. Additionally, a chest CT scan can aid in identifying concealed lung issues, which may not be visible through a radiograph, and evaluate the gravity of a chest's wind-swept deformity [1]. Further, the recurrence of heart defects demands a comprehensive cardiac examination and EKG [6]. If there is a previous history of cardiac disease or a murmur is detected, it is best to consult a cardiologist to assess the current heart condition [6].

It is important to conduct such a comprehensive assessment in order to achieve the optimal outcome for the patient undergoing major surgeries like spinal fusion. This evaluation enables medical professionals to be aware of potential new medical issues and unique challenges that may arise during and after the procedure, such as elevated risks of infection, healing complications, and bleeding [1]. For those with a high risk of developing FLNB-related diseases due to family history, prenatal diagnosis by sonography plays a crucial role in disease management [12,13,25]. The diagnosis aids in preparing for the disease's potential complications at birth or the pregnancy's termination [12,13,25]. In cases where a diagnosis is late, cesarean delivery may be necessary to prevent birth trauma [25]. Even if parents choose not to terminate, prenatal assessments remain essential to identify potential skeletal deformities that could affect delivery [12]. Those diagnosed with Larsen syndrome require specialized care in a neonatal intensive care unit [25].

When dealing with individuals diagnosed with Larsen syndrome, it is essential to conduct thorough and consistent static and dynamic serial radiography to keep track of their condition [26]. It is generally advised to steer clear of conservative management before any clinical symptoms arise since it can be arduous to anticipate postoperative recovery. While vigilant monitoring may lead to an improvement in neurological impairments, it is imperative not to postpone surgical correction. Delaying treatment could put younger children at risk of experiencing prolonged neurological degeneration, particularly when they're susceptible to frequent falls [26]. The progression of the disease underscores the imperative for timely intervention. Authors have proposed a non-invasive course of action that involves continual cervical traction and spinal column bracing for individuals born with severe disabilities [26]. Despite observing advancements in both respiratory and motor capacities, the researchers surmised that surgery might become necessary in the future [26]. As stated earlier, those with Larsen syndrome frequently encounter a myriad of interrelated skeletal irregularities [12]. Principal techniques to address these matters consist of orthopedic procedures and appropriate orthosis to rectify anomalies such as scoliosis, cervical spine instability, joint dislocations, and clubfoot [8,12].

The management of Larsen syndrome through surgery presents a significant challenge for spine surgeons, particularly in the cervical spine, where the condition tends to manifest most severely than in the thoracic or lumbar segments. As such, this area requires meticulous attention and long-term follow-up [26]. Prioritizing arthrodesis for cervical kyphosis in the sequence of orthopedic operations is essential [9,16]. In addressing cervical spine kyphosis and subluxation, it is crucial to perform fusion before addressing scoliosis, as stabilizing the kyphosis is integral to the overall management of the condition [1,9,27].

The treatment options for cervical displacement in Larsen syndrome involve numerous approaches, including anterior, posterior, and combined methods with 360° fusion [2]. MRI imaging may be employed to determine the urgency of stabilization [9]. The most appropriate course of action will depend on numerous factors, including the syndrome's specific presentation, the severity of the disease, and the age of the patient [8,10]. Posterior spinal fusion is only suggested for those presenting with mild and flexible cervical kyphosis [4,10,13,23,27,28]. For patients with severe or rigid kyphotic deformity who frequently develop myelopathy, anterior decompression and circumferential arthrodesis are the preferred option [4,10,13,23,27,28]. Young patients with small, hypoplastic, bullet-shaped vertebrae should not undergo anterior spinal fusion alone due to the considerable risk of spinal cord injury during decompression and difficulty stabilizing the reconstructed cervical spine. This process causes a halt in anterior growth and minimizes correction of the kyphosis [4,10,27,28]. Henceforth, it is imperative to conduct a prompt screening for cervical kyphosis in all Larsen syndrome patients upon diagnosis and consider posterior fusion a feasible option if detected early [10,24,27]. However, it is worth noting that certain medical experts suggest that in cases of severe kyphotic deformities, anterior surgery via a lateral approach may potentially provide better visualization and a safer cervical cord decompression. Nonetheless, such a procedure can carry various risks, including the possibility of retraction of the carotid sheath and release of the vertebral artery [27,28]. Alternatively, some medical professionals recommend utilizing the extended trans-oral route as a safer option, particularly for experienced surgeons specializing in craniovertebral junction surgeries [28]. Moreover, the option of single-stage 360° fixation is available, comprising corpectomy with implant or graft anteriorly alongside C1/occipital plate to C6 fixation [4]. This alternative is frequently chosen as it can be tailored to the degree of the kyphotic deformity or the presence of symptoms [4]. While there is an ongoing debate about which corridor should be prioritized in the 360° approach, it has been suggested that a reasonable strategy is to perform posterior arthrodesis using either segmental spinal instrumentation or placing pedicle screws after anterior spinal decompression [28]. Following posterior, anterior, or circumferential fusion, the cervical spine must be immobilized with a halo vest [10,26,27], preventing children from suffering significant neurological deficits following minor traumas such as falls [26].

Various surgical methods can be utilized to achieve arthrodesis in affected cervical spine segments. These methods can include atlantoaxial fusion or occipitocervical fusion with semirigid fixation, which can involve wire or cable constructs like Brooks, modified Brooks, and interspinous techniques, as well as the utilization of autologous rib or iliac crest grafts [10]. Semirigid constructs generally require postoperative halo immobilization. Alternatively, more rigid techniques like C1 to C2 fixation with transarticular screws or plate/rod and screw fixation can be opted for to avoid postoperative halo care [10]. However, for infants and young children with Larsen syndrome who have deficient skeletal structure and bone quality, these approaches can be challenging or risky. Therefore, the utilization of such structures necessitates vigilance when situating screws [10]. Moreover, proper positioning of the patient, with a radiographic affirmation of the reduction of flexion instability, and careful instrumentation are essential surgical aspects aimed at minimizing the risk of a neurological defect. It is crucial to preplan for decompression in case of instability that cannot be reduced. Adaptation of adult instrumentation to suit smaller patients may foster enhanced healing [10].

As previously stated, it is crucial that the surgical approach and timing of the procedure be customized to each patient based on their age and symptoms [16]. Performing fusion on young children under one can pose significant challenges. Thus bracing may be applied until surgery can be scheduled after 18 months of age [9]. Infants who undergo posterior cervical fusion at such a young age have a higher risk of developing pseudoarthrosis and experiencing failure [9]. Delaying posterior cervical fusion until roughly 18 months of age can result in a greater fusion rate and correction of the defect through continued anterior growth while having a stable posterior tether [9]. It is recommended that young patients with cervical instability undergo bracing until they are able to receive surgical correction, as the risk of falls can be significant in these individuals [24,26]. However, there is ongoing debate regarding the appropriate timing of bracing prior to surgical intervention in patients with progressive kyphosis [26]. In cases of adults with long-standing kyphotic deformities, correcting such deformities is generally unnecessary, as they tend to fuse naturally, and progress is unlikely [22]. Instead, excessive stress may compress the spinal cord in adjacent levels, making decision-making for management challenging. Surgical intervention is typically only required for the more heavily compressed adjacent level [22].

There has been deliberation surrounding the appropriate timing of surgery in consideration of the presence of symptoms [2,4,16]. Research has indicated that performing preventative fusion in patients without symptoms yields more favorable neurological outcomes compared to waiting until neural compromise occurs [2,4]. As a result, many experts advocate for surgical intervention at the point of diagnosis, even if there are no neurological deficits, as a means of preventing the detrimental effects of minor trauma. This is due to the fact that numerous cases in the literature have documented the sudden onset of quadriparesis following a fall [2,4]. The methodology used to correct complex vertebral displacements is contingent upon the nature of the displacement and may necessitate the employment of anterior, posterior, or combined approaches [2].

Larsen syndrome may result in an early onset of scoliosis, which may become inflexible and require immediate action [1]. In the past, early-onset scoliosis was dealt with using anterior and posterior surgical techniques that included correction, fixation, and bone graft fusion [12]. However, more recently, measures such as bracing, casting, and growth-friendly surgeries have been created to mitigate the further progression of the condition, enhance both spinal and thoracic growth, and safeguard lung function and development [12]. Those diagnosed with Larsen syndrome who have progressive scoliosis and show thoracic malformations, such as thoracic insufficiency syndrome, should consider surgical procedures that conserve growth, particularly if they are still in their skeletal developmental years [1]. Such procedures include VEPTR (Vertical Expandable Prosthetic Titanium Rib) expansion thoracoplasty and growing rods, which can be utilized to remedy a significant thoracic deformity in the transverse plane with a stiff chest wall, as well as mobile chest walls with minimal deformity, respectively [1].

When it comes to the challenging combination of hip dislocation and scoliosis, surgical treatment can be complex due to the impact of hip flexion contracture on sagittal spinal alignment. According to research, individuals who have experienced hip dislocations, especially those that are bilateral, tend to exhibit greater lumbosacral and lordotic lumbar spine angles [29]. Among clinicians, there is an ongoing debate over whether to prioritize treatment of the spine or the hip in such cases. However, based on the cases documented in the literature, priority should be given to scoliosis surgery [29]. This is due to several factors, including the unpredictable positioning of the pelvis following spinal fusion and the challenges in creating a surgical plan for the hips in the presence of significant spinal deformities and unusual muscle forces [29]. Ultimately, any surgical treatment plan must take into account the intricate relationship between scoliosis and hip dislocation.

The sternal splitting technique is a highly efficacious means of accessing the cervicothoracic junction in pediatric Larsen syndrome patients presenting with cervical kyphosis or scoliosis [30]. This surgical method is intricate and invasive, requiring the assistance of a cardiothoracic surgeon to ensure patient safety. Its primary objective is to stabilize spinal segments and arrest the progression of the deformities. However, as with any surgical intervention, it carries potential risks of complications, such as damage to the recurrent laryngeal nerve, pericardium, and esophagus. Due to these inherent hazards, this approach is restricted to rare cases where other alternatives have proved ineffective, and the patient's neurological health necessitates conservation [30].

The recuperation of the patient following surgery relies not only on cervical involvement but also on the existence of joint pathologies in regions such as the knee, ankle, or hip, as well as defects in other organs/systems [4]. Noteworthy joint dislocation typically calls for operative correction and bracing, whereas the treatment for clubfoot calls for a combination of conservative therapy and soft tissue and bone operations [12]. The most effective way to deal with cleft palate and hearing loss is through the utilization of multidisciplinary teams [13]. Lastly, pulmonary medical attention is of utmost importance for Larsen syndrome patients with restrictive lung disease [1].

Patients diagnosed with Larsen syndrome may present anesthesiological challenges due to their musculoskeletal, respiratory, cardiac, and neurological issues, necessitating recurrent anesthetic care [5,6]. Anesthesiologists must consider the life-threatening airway problems children with Larsen syndrome may experience. Upper respiratory illnesses or postoperative endotracheal intubation can exacerbate these problems by causing edema and respiratory compromise [5]. Moreover, postoperative respiratory issues can be worsened by bronchomalacia, which may lead to atelectasis and pneumonia [5]. Given these challenges, the underlying airway defects associated with Larsen syndrome are of paramount significance to the anesthesiologist in avoiding respiratory complications. Airway malacia is a common occurrence and may be a risk factor for perioperative respiratory compromise. The malacia can also affect the distal airways, impeding the effectiveness of tracheostomy in numerous scenarios [5]. During the perioperative period, it is recommended that aggressive pulmonary toilet be administered, which includes chest physiotherapy and mechanical ventilation if necessary. In cases of laryngomalacia, tracheostomy may be helpful, but distal bronchomalacia may require positive end-expiratory pressure (PEEP) and other treatments. Due to the high probability of respiratory and airway complications, patients should be closely monitored in an intensive care unit following surgery [5]. Additionally, great care must be taken when performing airway instrumentation on patients with spine abnormalities such as cervical kyphosis, which should be treated as if they have experienced traumatic cervical spine injury [5]. Possible airway management options for such patients may include oral endotracheal intubation with in-line axial traction, cervical stabilization, or fiberoptic techniques [5,6]. It is recommended that fragile conditions such as skeletal deformities, contractures, and cervical instability be handled with delicacy and precise positioning [6]. To ensure spinal cord integrity during tracheal intubation and positioning, early establishment of SSEP monitoring is suggested as it provides an important baseline and critical information [6]. Caution must be exercised when administering depolarizing neuromuscular blocking agents such as succinylcholine in patients with cervical instability and cord myelopathy who may have abdominal/intercostal muscle weakness due to the risk of hyperkalemia [6]. For patients with laryngotracheomalacia, it is preferred to use anesthetic agents with faster induction and recovery [13].

## Conclusions

Larsen syndrome is a rare congenital disorder that presents with joint dislocations and spine deformities. Effective management of this condition requires a comprehensive, interdisciplinary approach involving consultation with medical and surgical specialists and ongoing evaluation. Addressing cervical kyphosis is a primary objective in treating patients with Larsen syndrome. Various surgical interventions, including posterior spinal fusion, anterior decompression and circumferential arthrodesis, as well as growth-preserving techniques, may be employed based on the unique characteristics and severity of the disease. To achieve optimal outcomes, meticulous preoperative assessment and postoperative joint care, along with careful attention to detail and family/caregiver education, are essential.
